# Microbial community succession in response to sludge composting efficiency and heavy metal detoxification during municipal sludge composting

**DOI:** 10.3389/fmicb.2022.1015949

**Published:** 2022-10-05

**Authors:** Weijiang Han, Shuona Chen, Xiao Tan, Xin Li, Hua Pan, Peijian Ma, Zhihua Wu, Qilai Xie

**Affiliations:** ^1^College of Natural Resources and Environment, South China Agricultural University, Guangzhou, China; ^2^South China Institute of Environmental Sciences, Ministry of Ecology and Environment, Guangzhou, China; ^3^Guangdong Provincial Key Laboratory of Agricultural and Rural Pollution Abatement and Environmental Safety, Guangzhou, China; ^4^Nanhai Branch of Foshan Ecological Environment Bureau, Foshan, China; ^5^Qingyuan Solid Waste Treatment Center, Qingyuan, China

**Keywords:** municipal sludge composting, microbial community, heavy metal detoxification, physicochemical characteristics, metabolic pathway

## Abstract

This study researched microbial community succession in response to sludge composting efficiency and heavy metal detoxification during municipal sludge co-composting with spent mushroom and spent bleaching. The change law of key physicochemical properties, the heavy metals contents and forms during composting were analyzed, and the passivation of heavy metals after composting was explored. High-throughput sequencing was used to analyze the microbial community structure of treat 2 during composting, and the correlation analysis of microbial community structure with heavy metal contents and forms were carried out. The results showed that the sludge of each treatment reached composting maturity after 26 days of composting. Organic matter content, electrical conductivity, pH and seed germination index of treat 2 were all in line with the standard limit of agricultural sludge. Because of the presence of compost bacteria addition, the passivating heavy metals performance of treat 2 satisfied the standard limit of agricultural sludge after composting, which was superior to that of treat 1 and treat 3. The diversity of microbial communities in treat 2 decreased during composting. Extensive bacteria such as *Bacillus*, *Geobacter*, *Lactobacillus*, and *Pseudomonas*, which possessed the abilities of heavy metal passivation and organic oxidizing, were dominant in treat 2 during the heating stage. However, as composting proceeded, *Tuberibacillus* with ability of organic oxidizing gradually became the most dominant species at the thermophilic and cooling stages. Changes in microbial function varied from changes of microbial community in treat 2, subsequently affected the performances of heavy metal passivation and organic oxidizing during composting.

## Highlights

- The sludge reached maturity after 26 days of co-composting with spent mushroom and spent bleaching.
- The effect of passivating heavy metals by treat 2 satisfied agricultural standard.
- The diversity of microbial community in treat 2 decreased during composting.
- Microbes with abilities of heavy metal passivation and organic oxidizing were enriched.

## Introduction

With the rapid development of urbanization, and the improvement of sewage treatment rate in China, the output quantity of municipal sludge has increased rapidly (Chen Z. et al., 2021). Municipal sludge is an inevitable product of sewage treatment plants and sewage treatment. However, the composition of municipal sludge is usually very complex. Municipal sludge principally contains a large amount of salt, organic matter (OM) and heavy metals (such as Cr, Cu, Zn, As, Hg, Cd, etc.). It also contains residual refractory organic contaminants and various species of bacteria (Zhou et al., 2020). These components could cause municipal sludge and agricultural soil to exhibit certain biological toxicity (Xu et al., 2020, 2021). If the sludge enters the environment without correct treatment, it will directly result in secondary pollution of the water and the atmosphere. Consequently, it will not only reduce the capacity of sewage treatment systems, but also pose a serious threat to human living environment and health (Liao et al., 2018). It has been suggested that only 31–36% of sludge produced by municipal sewage treatment plants in China was treated with harmless treatment and disposal in 2015 (Wang et al., 2015). Compared with the increasing amount of sludge, the current rate of harmless treatment and disposal of sludge is too low to avoid serious consequences. In addition, the heavy metals in sewage sludge will cause serious pollution without the correct disposal and utilization method, which limits its application on soil nutrient (Liu et al., 2014; Chen R. et al., 2021). What’s more, the difficulty to simultaneously passivate heavy metals and enrich nutrients still existed in sewage sludge (Lu et al., 2021). Therefore, it is a matter of utmost urgency to find a proper way to reduce, render harmless and recycle sludge with complex components and huge output. Public entities in China must prepare to solve this problem.

Aerobic composting is an effective method for treating organic solid waste (Zhou et al., 2020). Composting mainly relies on the metabolic activities of various microorganisms from nature, such as *bacteria*, *actinomycetes*, *fungi*, and *protozoa*. It converts some of the available OM into inorganic matter with a simple structure and releases heat. Simultaneously, the other portion of the OM is transformed into new cytoplasm, which allows microorganisms to grow and reproduce continuously, produces more organisms and continuously degrades the OM in organic solid waste (Zorpas et al., 2017). Some studies have shown that composting can achieve heavy metal passivation and the removal of refractory organics (Liao et al., 2018; Chen R. et al., 2021). Previous researches have demonstrated that two key control parameters, initial C/N ratio and bulking agent type, can significantly affect heavy metal immobilization during composting (Guo et al., 2022). However, from an intrinsic perspective, heavy metal immobilization is highly associated with microbial activities since composting is essentially a matter transformation process mainly through the metabolism of bacteria and fungi (Mao et al., 2019). Microorganisms have an important role in the composting process. Taking microorganisms as the executor to realize the resource, harmless and reduction of solid waste has become a research hotspot for sludge disposal. Modern molecular biology technology is convenient for research regarding composting microorganisms (Wang et al., 2015). The detoxification of heavy metals and the degradation of OM in compost can be promoted by the addition of appropriate microbial community (Chen Z. et al., 2021). The efficiency and quality of the composting process depend on the diversity of the microbial flora in the compost. In the microbial composting system, there is not a clear understanding regarding the correlation among the microbial community structure, the morphological changes of heavy metals in the composting process. This is consequence of the composting system’s complexity.

Due to the property of municipal sludge itself is usually difficult to meet the conditions required by sludge composting, it is necessary to add auxiliary materials to reduce sludge moisture content (MC) and improve porosity, so as to meet the needs of microbial growth, thus speeding up the composting process, improving the composting effect and promoting the detoxification of composting to heavy metals. Spent mushroom is rich in OM and nutrients, and has a fluffy structure, so it has been used as one of the conditioner of sludge compost to improve the fertilizer and structural porosity of compost (Jordan et al., 2008). Spent bleaching earth is a by-product of refining process of oil factory. It has a large yield and high OM content of about 50%, but low heavy metal content. It can improve the odor generation by using it in compost (Ding et al., 2019). Therefore, spent mushroom and spent bleaching earth can improve the potential of composting process due to their high contents of OM and nutrients, and fluffy structure. Using spent mushroom and spent bleaching earth as auxiliary materials in urban sludge composting can effectively improve the composting effect and realize the resource utilization of spent mushroom and spent bleaching earth.

Based on the above analysis, a high-temperature aerobic composting process was proposed in this study to explore the effects of composting bacterial agent and auxiliary materials on the physicochemical properties of municipal sludge composting. Residual sludge from urban sewage treatment plants, spent mushroom and spent bleaching earth were utilized in the composting, and the initial ratio of sludge, spent mushroom, and spent bleaching earth in the compost was set as 12:10:3. In addition, the passivation of heavy metals from municipal sludge after composting was investigated. Samples were collected at different stages of composting, and high-throughput sequencing was used as a measure to analyze the microbial community structure in the composting process, so as to research the law of microbial community succession in the composting process of municipal sludge. Accordingly, the change rule of key physicochemical properties, the heavy metals contents and forms in the composting process were analyzed, and the correlation analysis of microbial community structure with heavy metal contents and forms were carried out to explore the influence of changes in microbial community structure of the compost on the passivation effect of heavy metals. An objective of the current work to provide a basic reference for the technological development of heavy metal passivation in municipal sludge composting from the perspective of microbial community succession.

## Materials and methods

### Experimental materials

#### Compost raw and auxiliary materials

Raw and auxiliary compost materials include fresh sludge, spent mushroom, and spent bleaching earth. Municipal sludge was taken from a sewage treatment plant in Dongguan City and used as the compost raw materials for this project after the drying treatment. Spent mushroom and spent bleaching earth were used as compost auxiliary materials. The basic properties and the heavy metals contents of raw and auxiliary materials are as shown in Supplementary material.

#### Microbial inoculum

The microbial inoculum adding to compost, which is recorded as M1, is a complex lively bacterial preparation obtained and preserved by preliminary screening in the laboratory. The content of microbial inoculum M1 adding to compost in this study was 1%.

### Compost treatment method

Three composts were configured according to the addition of microbial inoculums to investigate the effects of microbial inoculums on the detoxification of heavy metals during 26 days composting process. The initial ratio of sludge, spent mushroom, and spent bleaching earth in the compost was set as 12:10:3. After the raw and auxiliary materials of the experimental compost were intensive mixed, they were stacked into a conical pile about 0.6 m high with a diameter of about 1.5 m. The pile was placed under the greenhouse of the workshop of the composting plant and was composted by the method of high temperature aerobic static ventilation without adding any ventilation equipment. During this period, the pile was operated with artificial turning. In the early stage of compost fermentation, when the temperature was less than 45°C, the compost was not turned over. When the temperature exceeded 45°C, the piles were turned once a day in the morning. If the temperature of the piles rose slowly, the piles were turned over immediately. The settings for adding microbial inoculum to the piles are listed in Table 1.

**TABLE 1:** Compost method setting.

Items	Compost setting
treat 1	Raw sludge
treat 2	Sludge: spent mushroom: spent bleaching earth = 12:10:3, with 1% M1 addition
treat 3	Sludge: spent mushroom: spent bleaching earth = 12:10:3, without M1 addition

### Sample collection and speciation analysis of heavy metals

Based on the composting temperature trend, the composting process was divided into different stages. Samples were collected at the pre-composting stage, the heating stage, the high-temperature stage and the cooling stage. Temperature sensors were used to measure the temperature of the upper, middle and lower parts of the reactor each morning and afternoon. The measured temperature of the day was the average value. Simultaneously, the ambient temperature was recorded.

During the composting process, the compost was turned over and stirred once every 3 days. After turning the piles and stirring evenly, samples were taken from five points in the front, back, left, right and center of the pile, and mixed equally into one sample. Then, we obtained a sample of 2 kg based on quartering after spreading and dividing it into three parts. One fresh sample was used for the determination of pH, conductivity and MC (Wang et al., 2021); one air-dried, crushed and sieved for the determination of heavy metals; one sample was placed in a kraft paper envelope and stored in a sealed bag in a cryogenic refrigerator for the determination of OM. To study the species distribution of heavy metals in the municipal sludge, a modified BCR (the Community Bureau of Reference) three-step sequential extraction method was used to determine the speciation and content of heavy metals in the samples (Rauret et al., 2000; Zhou et al., 2020). Using this method, the speciation of heavy metals can be divided into four forms: acid-exchangeable form, reducible form, oxidizable form and residual form. Finally, the samples in the temperature-increasing period, high-temperature period and mature period of treat 2 will be used for the determination of microbial community. They will be taken from the 3rd, 12th, and 24th days of the composting process, respectively. Each biological sample was subjected to three parallel experiments and named WN3d-1, WN3d-2, WN3d-3, WN12d-1, WN12d-2, WN12d-3, WN24d-1, WN24d-2, and WN24d-3.

### Method of high-throughput sequencing

Microbial communities of the 12 biosamples were sequenced. The microbial signature sequence of 16S rDNA (ribosomal feature coding sequence of prokaryotes) by PCR amplification was sequenced using high-throughput sequencing technology (Liang et al., 2021). A FastDNA™ SPIN Kit for Soil (MP Biomedicals, Strasbourg, France) was used to extract microbial DNA according to the manufacturer’s instructions. The extracted DNA products were analyzed using agarose gel electrophoresis. Then the V3 + V4 region of 16S rDNA was amplified using specific primers with barcode. The primers used were as follows: 341F: CCTACGGGGGGCWGCAG, 806R: GGACTACHVGGGTATCTAAT. A QuantiFluor™ fluorometer was used for the quantitative determination of the amplified products, which were cut into gels for recovery. The purified amplified products were mixed in equal quantities and sequenced by connecting the sequencing joints. The sequencing library was constructed according to Illumina’s instructions, and the PE250 mode of Hiseq2500 was used for sequencing. The original DNA sequences were filtered by Mothur (v1.39.1) to remove the chimera, and finally the majorized sequences were obtained. Uparse (usearch v9.2.64) was used to classify the sequences. The sample and species taxon sequence abundance matrix were constructed by calculating the sequence abundance of each sample and each taxon. Multiple sequences were clustered according to their distance. Clustering the sequences with 97% identity reads into operational taxonomic units (OTUs) was the default; the rarefaction curve was analyzed based on OTU. Finally, the Chao1 index, Coverage and Shannon diversity index were calculated to measure the diversity of the samples.

### Statistical analysis

All determination experiments were repeated for two-three instances, and the experimental data were expressed as the mean ± standard deviation. The differences among the data were tested using the Student’s *t*-test (*P* < 0.05).

## Results and discussion

### Changes in physicochemical properties during composting

**FIGURE 1:**
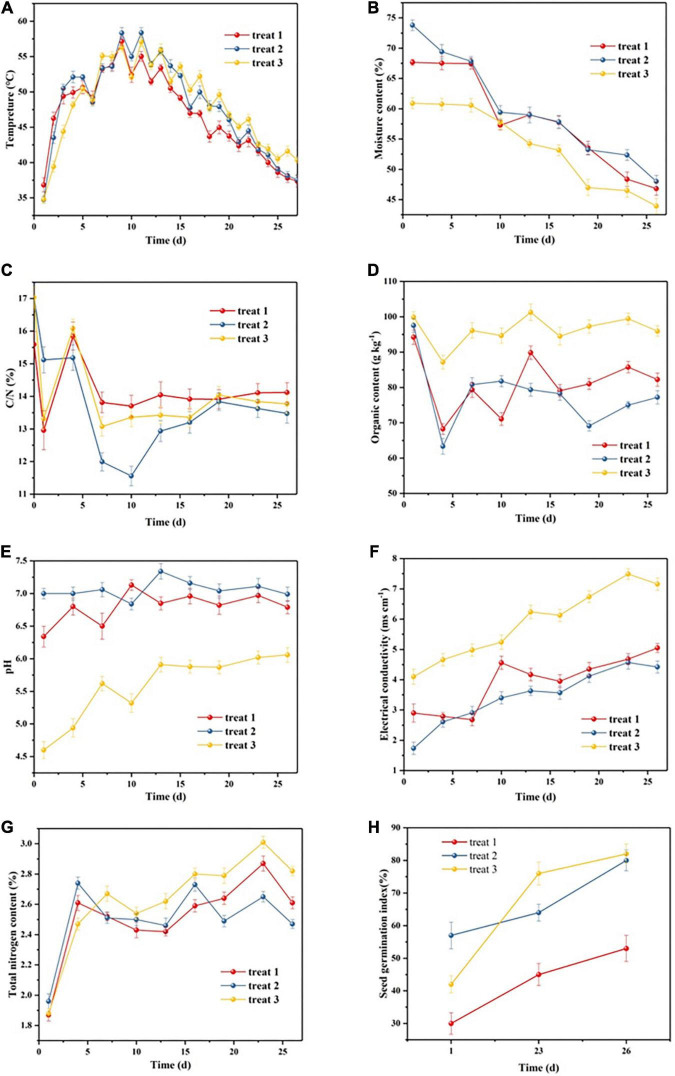
Changes in physicochemical characteristics of different piles during the composting process. **(A)** Temperature, **(B)** moisture content, **(C)** C/N, **(D)** organic matter content, **(E)** pH, **(F)** electrical conductivity, **(G)** total nitrogen content, and **(H)** seed germination index. Error bars represented standard deviation (*n* = 3).

The physicochemical properties of the different treatment groups are shown in Figure 1. During the composting process, the variation in temperature of all treatment groups showed a similar trend (Figure 1A), which was roughly divided into three stages: the heating stage (0–7 days), the thermophilic stage (7–15 days), and the cooling stage (15–26 days). This indicated that the number of microorganisms in all treatment groups rapidly increased and then contributed a lot of heat from degrading OMs, leading to rapid evaporation of water. Compared with other treatment groups, treat 1 showed lower temperature during the composting process, which might be a result of no special compost bacteria and auxiliary materials added to the pile of treat 1. In addition, the native microbial activity of treat 1 was obviously inhibited by heavy metals (Zhou et al., 2020). The temperature of treat 2 and treat 3 increased more quickly compared with treat 1, indicating that auxiliary materials could enhance the nutrient conditions for the composting process and the addition of composting microbes promoted the composting effect. All treatment groups rapidly reached the greatest temperatures (>50°C) at day 7, which remained above 55°C for more than 6 days, and then decreased gradually. It was reported that as the fermentation temperature was maintained 55°C for more than 3 days, the majority of pathogenic microorganisms could be inactivated, and then the compost products achieved the standard for harmless levels (Ezzariai et al., 2018; Awasthi et al., 2019).

As shown in Figure 1B, the initial MCs of all treatment groups were adjusted to be between 60 and 75% to create suitable living conditions for microorganisms. During the composting process, the MC of all treatment groups exhibited a continuous downward trend, but there were significant differences between the three treatment groups. As demonstrated in Figure 1B, after composting for 26 days, the MCs of treat 1, treat 2, and treat 3 decreased to 46.82, 48.00, and 43.94%, respectively. These values decreased by approximately 30.81, 34.96, and 27.85% of the initial MCs, respectively. By comparison, it was found that the water volatilization in treat 2 was the most rapid, resulting in the best minimization effect in treat 2.

During the composting process, carbon and nitrogen sources are essential nutrients for microbial activities, so C/N is the key factor affecting composting process and product quality. C/N can directly reflect the impact of crops in composting, and too high or too low C/N will affect the growth of composting microorganisms. As shown in Figure 1C, the C/N values of the three treatment groups at the initial composting period were around 16, which were mainly due to the high ratio of sludge in composting raw materials. The C/N values of the three treatments all showed a trend of falling first and then rising. Compared with treat 1 and treat 3, the C/N value decreased most rapidly in treat 2, which was dropped to the lowest value of 11.56, but then began to rise gradually and tended to balance. This indicated that in the first 10 days carbon source of treat 2 was rapidly degraded during the composting process, while utilization rate of nitrogen source was slower. However, the utilization rate of nitrogen source was accelerated in the follow 16 days, and the utilization rate of nitrogen source was higher than that of carbon source, therefore, C/N rose, and tended to balance with composting processing. Since no additional compost microbes were added to treat 1 and treat 3, there might be the main reason for the fastest C/N decrease in treat 2. Thus, it could be seen that the C/N ratio of treat 2 accelerated the degradation of OM during composting compared with treat 1 and treat 3, which indicated that treat 2 could not only accelerate the degradation of organic carbon in sludge compost, but also had a certain effect on nitrogen retention.

Composting is a process of humification and mineralization of OM. Therefore, as shown in Figure 1D, during the composting process, the OM content of each treatment exhibited a declining trend. The initial OM contents of treat 1, treat 2, and treat 3 were 94.22, 97.52, and 99.91 g kg^–1^, respectively. After composting, the OM contents of treat 1, treat 2, and treat 3 were 82.24, 77.21, and 95.54 g kg^–1^, respectively. These decreased by 12.71, 18.77, and 4.37%, for treat 1, treat 2, and treat 3, respectively. It was worth noting that at the end of composting, the OM content of each treatment showed an increasing trend, which might be due to the decrease in water content of the three piles and the occurrence of OM concentration phenomenon with the composting process (Mkaa et al., 2020).

Generally, the pH value affects microbial activity and the mobility of heavy metals, and the optimal pH of composting is of 6.5∼8.0. As shown in Figure 1E, except for treat 1 and treat 3, the pH of treat 2 remained between 6.5 and 7.5 during the composting process, which was suitable for the growth and reproduction of compost microorganisms (Czeka et al., 2016). The results demonstrated that the pH of treat 2 remained stable between 6.5 and 7.5, which met the physicochemical properties of organic fertilizer (Wang et al., 2013).

Electrical conductivity (EC) reflects the amount of soluble salt in compost. During composting, complex organic substrates were biodegraded into dissolved components, leading to a gradual increase in the EC of each treatment group during the composting process. As shown in Figure 1F, the EC trends between treat 1 and treat 2 showed little difference. The initial ECs of treat 1 and treat 2 were in the range of 1.8∼3.0 ms cm^–1^, while increased to the range of 4.0∼4.6 ms cm^–1^ at the end of composting. Among the three treatments, the EC trend of treat 3 was the highest during the whole composting process, rising from about 4.0 to about 7.1 ms cm^–1^, but less than 9 ms cm^–1^ (Mkaa et al., 2020), indicating that the sludge of each treatment after composting would be beneficial to soil utilization.

As shown in Figure 1G, during the composting process, the total nitrogen (TN) content of all treatment groups initially increased, subsequently decreased, and then increased, prior to finally decreasing. At the initial stage of composting, ammonia volatilization was reduced owing to the insignificant microbial ammoniation and the continuous decrease in water content. The TN content of each treatment increased with the concentration effect of composting. In the second phase, the increase in TN might be a result of the obvious decrease in water content in the piles during the thermophilic stage, leading to the concentration effect of TN (Li et al., 2015). The decrease in TN in each pile was due to the strong metabolic activity of microorganisms, which decomposed organic nitrogen into ammonia and volatilized it into air. The results indicated that all treatment groups had an effect on nitrogen preservation after composting.

Seed germination index (GI) is an important index to judge the compost maturity and can be used to directly and effectively detect the toxicity of compost products. As shown in Figure 1H, the GI value of treat 1 increased from 0.3 on day 1 to 0.53 on day 26. After mixing with auxiliary materials and adding composting bacteria, the GI values of treat 2 and treat 3 were ranged from 0.42 to 0.82 and 0.57 to 0.80, respectively. It could be seen that both GI of treat 2 and treat 3 was above 0.80, indicating that auxiliary materials and composting microorganisms addition in the compost could effectively reduce sludge toxicity.

### Performance of heavy metal passivation during the composting process

Table 2 displays the changes of the total amount of heavy metals in the different treatments before and after composting. The total amount of heavy metals in the different treatments decreased after composting. As obvious water stains were observed at the bottom of the piles during the composting process, it could be supposed that leachate with some heavy metals exited from the bottom of the piles, which might be one of the reasons for the decrease in the total amount of heavy metals in each pile after composting. The contents of Cu, Cd, and Zn in treat 1 decreased by 16.05, 42.40, and 3.94%, respectively. In treat 2, the contents of Cu, Cd, and Zn decreased by 35.44, 35.71, and 0.10%, respectively. In treat 3, the contents of Cu, Cd, and Zn decreased by 26.54, 36.78, and 8.19%, respectively. It might be observed that most of the Cu and Cd contents in treat 2 and treat 3 decreased, which might be caused by the loss of more filtrate in the pile after the addition of the composting bacterial agent. As indicated by Table 2 and Figure 1F, the content of Cu, Cd, and Zn in treat 1 at the end of composting were 572.7, 0.65, and 414.2 mg kg^–1^, respectively. The Cu content exceeded the agricultural standard of 500 mg kg^–1^ (pH > 6.5), while the other two heavy metals satisfied the concentration standards prescribed in the Pollutants Control Standard for Agricultural Sludge (GB4284-2018) because of their low initial values (Zhang et al., 2016). At the end of composting, the contents of Cu, Cd, and Zn in treat 2 were 286.4, 0.54, and 294.8 mg kg^–1^, respectively, so the contents of the three heavy metals satisfied the concentration limit prescribed in the Pollutants Control Standard for Agricultural Sludge (GB4284-2018) while pH > 6.5 (Zhang et al., 2016). The contents of Cu, Cd, and Zn in treat 3 were 320.6, 0.55, and 314.8 mg kg^–1^, respectively, indicating that Cu exceeded the limit concentration of 250 mg kg^–1^ in agricultural standard while pH < 6.5. However, Cd and Zn concentrations satisfied the limit concentration prescribed in the Pollutants Control Standard for Agricultural Sludge (GB4284-2018) (Zhang et al., 2016). Overall, only the composting products of treat 2 met the requirements for safe agricultural use after composting.

**TABLE 2:** Changes of heavy metals in municipal sludge composting before and after treatment.

Items		Cu (mg kg^–1^)	Cd (mg kg^–1^)	Zn (mg kg^–1^)	pH
Treat 1	Initial	682.2 ± 7.7	1.17 ± 0.10	431.2 ± 8.8	6.34
	Final	572.7 ± 7.0	0.65 ± 0.14	414.2 ± 6.4	6.79
Treat 2	Initial	443.6 ± 6.8	0.84 ± 0.09	295.1 ± 6.7	7.00
	Final	286.4 ± 7.4	0.54 ± 0.04	294.8 ± 5.4	6.99
Treat 3	Initial	436.4 ± 6.5	0.87 ± 0.11	342.9 ± 5.3	4.60
	Final	320.6 ± 6.6	0.55 ± 0.09	314.8 ± 6.0	6.06
National agricultural standard for sludge (GB 4284-84) (pH < 6.5)		<250	<5	<500	/
National agricultural standard for sludge (GB 4284-84) (pH > 6.5)		<500	<20	<1000	/

**FIGURE 2:**
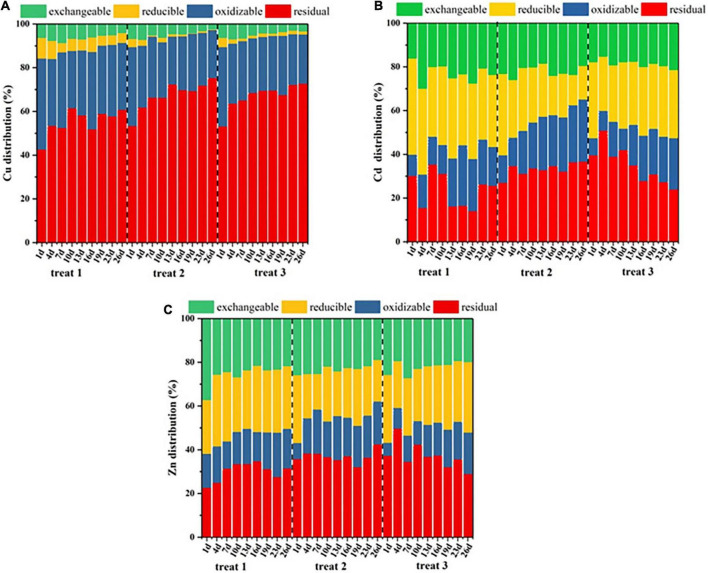
Changes in **(A)** Cu distribution, **(B)** Cd distribution, and **(C)** Zn distribution during the composting process.

The bioavailability of heavy metals is closely related to their forms. In this study, the improved BCR fractional extraction method was used to determine the different forms of heavy metals, such as acid exchangeable, reducible, oxidizable and residual fractions. As shown in Figure 2, there was a passivation effect on the Cu element in treat 2 and treat 3 after composting, which was mainly reflected in the contents of refractory forms, including residual and oxidizable fractions. As the composting process progressed, the different forms of the Cu distribution in treat 1 did not change significantly, and the percentage of refractory forms increased by only 3.77% compared with that before composting. However, the contents of the Cu refractory form in treat 2 and treat 3 increased by 7.80 and 6.24%, respectively. It could be seen that among the three treatments, the largest proportion of Cu in treat 2 had been transformed from easy migration form to refractory form, showing the best passivation effect after composting. Cd in the composting mainly existed as residual, oxidizable, reducible and acid exchangeable fractions. During the process of composting, the content of residual Cd in treat 1 was not obvious changing in treat 1. During the process of composting, the change trends of residual Cd and oxidizable Cd in treat 2 were gradually increased, the content of residual Cd in treat 2 increased from 27.20 to 39.83%, with an increment of 13.63%. Similarly, the oxidizable Cd content in treat 2 increased from 12.60 to 30.70%, with an increase of 18.10%. Therefore, the refractory Cd content increased 31.73%. The content of residual Cd was decreased from 39.73 to 24.16%, while the content of oxidizable Cd was increased from 7.87 to 23.45% after composting. The total content of refractory Cd was decreased by 0.71%. In conclusion, during the composting process, the passivation effect of Cd in treat 1 was not obvious. Compared with the composting process of treat 2 and treat 3, it was found that the additional bacterial agent to the composting had a superior passivation effect on Cd than no bacterial agent to the composting. The content of residual Zn increased from 22.71 to 31.61% with an increment of 8.90%, and the content of oxidizable Zn increased from 15.54 to 18.12% with an increment of 2.58%. Therefore, the content of refractory Zn was increased by 11.48%, and the total content of refractory Zn reached 49.72%. In the composting process of treat 2, different from that of treat 1, the content of residual Zn increased from 35.61 to 42.58% with an increment of 6.97%, while the content of oxidizable Zn only increased by 1.50%. Consequently, the total amount of refractory Zn reached 62.20%. After composting, the content of refractory Zn in treat 3 also showed a slight upward trend that increased from 43.32 to 48.02%, indicating that the passivation effect on Zn was obvious, among which the composting effect of treat 2 was superior to the other treatments. Overall, after composting, the effects of Cu, Cd, and Zn passivation in treat 2 were superior to those in treat 1 and treat 3. The results showed that the addition of composting bacteria was an important reason to promote the passivation of heavy metals such as Cu, Cd, and Zn.

### Microbial community characteristics in treat 2 during composting

#### Diversity of microbial community during composting

Table 3 shows the alpha diversity indices of treat 2 biosamples including Chao1, Ace, Shannon, and Simpson during composting. As shown in Table 3, the coverage index of the treat 2 biosamples at all stages of composting were greater than 0.99, indicating that the sequencing results could better represent the real situation of microorganisms in the treat 2 biosamples (Chen et al., 2019). The Chao1 index and the ACE index were used to evaluate the ecological indices for estimating the total number of species during composting (Wang et al., 2016). As composting time progressed, the Chao1 index and the Ace index decreased from 3,263 and 3,309 on day 3 to 2,505 and 2,577 on day 24, respectively, suggesting that the alpha diversity of treat 2 biosamples decreased gradually during composting. The Shannon index and the Simpson index were important indices for estimating the diversity and richness of the microbial community distribution during composting. The larger the Shannon index, the higher the microbial community diversity of biosamples, and the larger the Simpson index, the lower the microbial community richness of biosamples (Vitali et al., 2016). The Shannon index with an average number of 8.06 at the early composting stage (WN3d) was greater than that with an average number of 4.99 at the late composting stage (WN24d), indicating that composting reduced the diversity of microbial community. Conversely, the Simpson index with an average of 0.86 at the early composting stage was significantly lower than that with an average of 0.99 at the late composting stage, indicating that composting reduced the richness of microbial community. The decrease in microbial diversity and richness might have been caused by the inactivation of microorganisms in a high temperature environment. In addition, it could be found from Table 3 that the average OTUs of WN3d was of the highest (2393), followed by WN12d (1883) and WN24d (1365). The above results indicated that the average OUTs decreased as composting proceeded.

**TABLE 3:** The alpha diversity of microbial communities in compost samples.

Sample	Chao1	Ace	Shannon	Simpson	OTUs	Coverage
WN3d-1	3265.19	3265.75	8.01	0.87	2589	0.993
WN3d-2	3313.55	3375.56	8.13	0.87	2647	0.992
WN3d-3	3212.77	3287.28	8.02	0.85	2530	0.992
WN12d-1	2934.22	2996.22	7.27	0.97	2208	0.992
WN12d-2	2900.83	2931.42	7.06	0.96	2146	0.993
WN12d-3	3220.07	3254.95	6.89	0.95	2208	0.991
WN24d-1	2561.70	2591.78	4.84	0.99	1679	0.992
WN24d-2	2442.90	2579.37	5.02	0.99	1663	0.993
WN24d-3	2512.55	2560.22	5.11	0.99	1727	0.993

**FIGURE 3:**
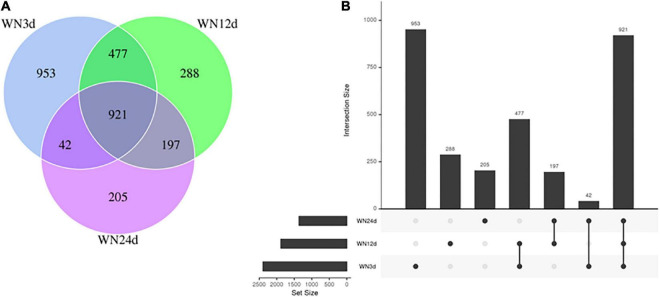
**(A)** Venn diagram of OUTs and **(B)** histogram of OUTs during the composting process.

The species distribution of microbial communities in different compost habitats was similar and specific to a certain extent. Based on the temperature, the nine compost biosamples could be divided into three groups including the heating group (WN3d), the thermophilic group (WN12d), and the cooling group (WN24d). These groups were used to perform cluster analysis on the composition of OTUs in the biosamples, to understand the common or unique information of OTUs between different biosamples. All OTUs with an average abundance greater than 1 in the comparison groups were selected for Venn diagram analysis (Figure 3). As shown in Figure 3A, WN3d, WN12d, and WN24d aggregations were filled with different colors, and the number of OTUs is marked in the diagram. Figure 3B shows the bar chart of the OTU statistics for each group. Each point represents a biosample group, and the line between the points represents the intersection of two points. As displayed in Figure 3B, the bar lengths between two connected points increase with the correlation between the two groups represented by the points. It might be observed that the shorter distance between WN3d and WN12d suggested a more similar OTU composition. The OTUs composition of WN12d and WN24d were of similar due to the shorter distance between WN12d and WN24d. However, there was a significant difference between WN3d and WN24d. The number of common OTUs in the biosamples of WN3d, WN12d, and WN24d was 921, which accounted for 16.33% of the total OTUs. The number of common OTUs between WN3d and WN12d was 1398, which accounted for 58.42 and 74.42% for WN3d and WN12d, respectively. The number of common OTUs between WN12d and WN24d was 1118, which accounted for 59.37 and 81.90% for WN12d and WN24d, respectively. Venn diagram analysis indicated that the compost temperature was the principal factor influencing the microbial community.

**FIGURE 4:**
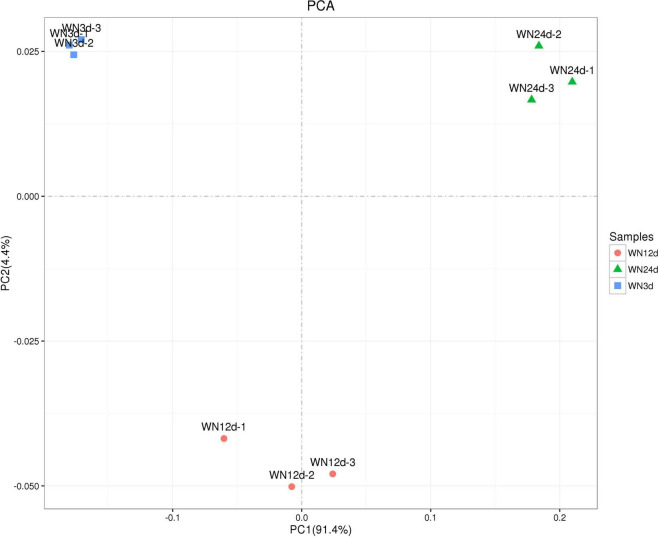
Principal component analysis (PCA) analysis on the similarity of bacterial communities during the composting process.

Principal component analysis (PCA) is commonly used to explore the similarities between different biosamples (Chen et al., 2020). As shown in Figure 4, PC1 and PC2 explaining the variance information accounted for 91.4 and 4.4% of the total variation, respectively. The greater the similarity between the compositions of biosamples, the smaller the distance reflected in the PCA diagram. In addition, the different biosamples might exhibit their respective aggregation distributions. The nine biosamples were divided into three groups (WN3d, WN12d, and WN24d) according to the distance between the biosamples. The PCA results showed that the biosamples at the same composting period shared more similarities, while the biosamples at different composting periods shared large differences. The cluster distance and PCA analysis indicated that the similarity in bacterial community composition was mainly influenced by the composting temperature.

#### Microbial community succession during the composting process

**FIGURE 5:**
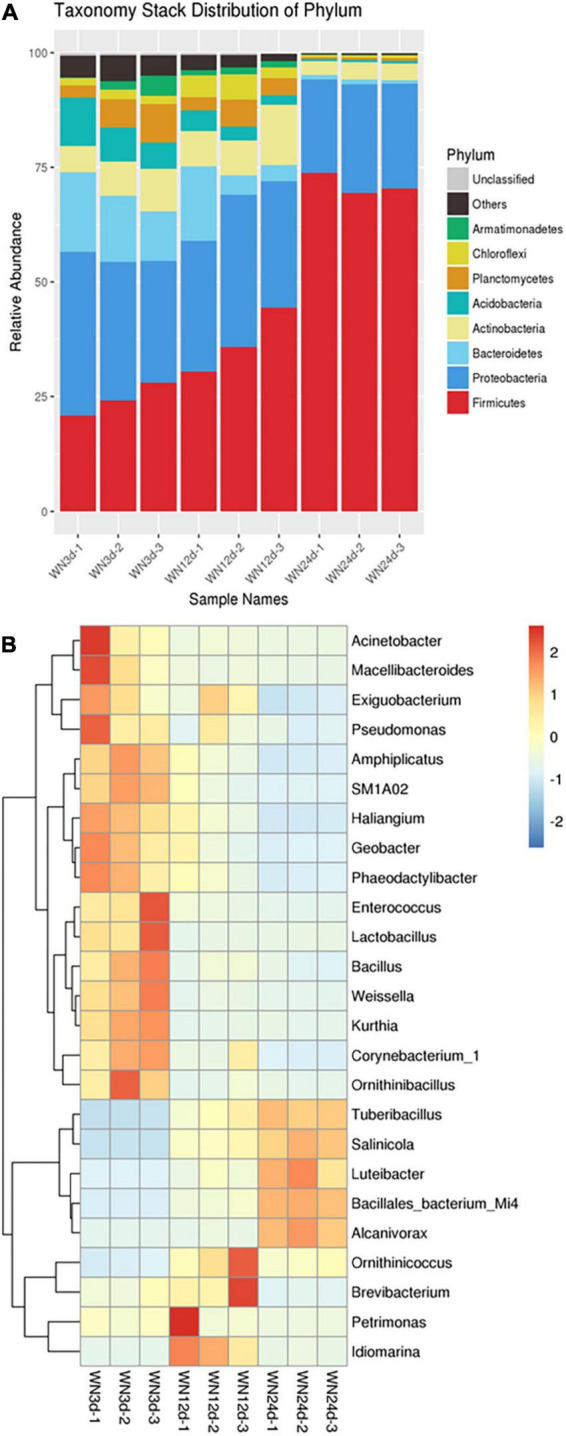
The distribution of microbial community at **(A)** phylum and **(B)** genus levels during the composting process.

Figure 5A reflects the changes in microbial community succession in treat 2 at the phylum level during the composting process. The main phyla in treat 2 during composting included *Firmicutes*, *Proteobacteria*, *Bacteroidetes*, *Actinobacteria*, *Acidobacteria*, *Planctomycetes*, *Chloroflexi*, and *Armatimonadetes*. Among them, *Proteobacteria* and *Firmicutes* were the two most dominant phyla in WN3d with relative abundances of 35.64 and 28.01%, respectively. Other phyla such as *Bacteroidetes*, *Actinobacteria*, *Armatimonadetes*, *Acidobacteria*, *Chloroflexi*, and *Planctomycetes* accounted for a certain proportion at the heating stage (WN3d). At the thermophilic stage (WN12d), the relative abundance of *Firmicutes* increased significantly, while the relative abundance of *Proteobacteria* decreased slightly. *Firmicutes* and *Proteobacteria* were still the two most dominant phyla at the thermophilic stage (WN12d) with relative abundances of 44.45 and 33.18%, respectively. At the cooling stage (WN24d), the relative abundance of *Firmicutes* rapidly increased to 73.81%, and *Firmicutes* became the dominant phylum. Other phyla such as *Proteobacteria*, *Actinobacteria*, *Bacteroidetes*, *Armatimonadetes*, *Acidobacteria*, *Chloroflexi*, and *Planctomycetes* decreased to varying degrees. At the initial stage of composting, *Proteobacteria* was the dominant phylum. With the progress of composting, the abundance of *Firmicutes* increased significantly and became the dominant phyla at the thermophilic stage and the cooling stage. The main reason for this might be that *Proteobacteria* possessed lower heat resistance than *Firmicutes*. Therefore, *Firmicutes* gradually replaced *Proteobacteria* and became the most dominant phylum during composting.

A heatmap diagram of microbial community distribution at the genus level is shown in Figure 5B. It was not difficult to observe that the dominant bacteria showed obvious succession in different stages of composting. At the heating stage (WN3d), the dominant genera mainly included *Weissella* (5.08%), *Corynebacterium*_1 (3.36%), *Amphiplicatus* (3.01%), *Enterococcus* (2.32%), *Bacillus* (2.22%), *Amphiplicatus* (3.36%), *Amphiplicatus* (2.32%), *Acinetobacter* (1.87%), *Macellibacteroides* (1.49%), *SM1A02* (1.49%), *Kurthia* (1.48%), *Geobacter* (1.28%), *Ornithinibacillus* (1.24%), *Lactobacillus* (0.93%), and *Pseudomonas* (0.82%), indicating the abundant community structure of treat 2 at the beginning of composting. It was also found that some common bacteria contributed to the passivation of heavy metals and removal of OM widely present in the heap compost. For example, *Bacillus* has a high affinity for metal binding and can adsorb and detoxify heavy metals in sewage and composting (Jacob et al., 2018). *Lactobacillus* is a lactic acid fermentation bacterium that may cause acidification in the presence of OM and is suitable for survival under acidic conditions (Na et al., 2021). *Geobacter* and *Pseudomonas* have good adsorption and reduction abilities for Cd, Ca, Zn, Cr, Mn, Pb, and other heavy metals (Fernández et al., 2018; Qiu et al., 2020). In addition, *Pseudomonas* was also the main degradation bacterium of OM (Qiu et al., 2021), which contributed significantly to the passivation of heavy metals and the removal of OM in treat 2. At the thermophilic stage (WN12d), the abundance of *Tuberibacillus* increased significantly and reaching 31.47%, subsequently *Tuberibacillus* became the most dominant bacterium in the high temperature period (Ding et al., 2019). According to literature reports, the removals of volatile organic sulfur compounds and organic acids were positively correlated with the bacterial activity of *Tuberibacillus* (Ding et al., 2019). Thus the enrichment of *Tuberibacillus* at the thermophilic stage was beneficial for the removal of OM. This might be one of the main reasons why C/N declined rapidly by treat 2 (Ding et al., 2019). The abundances of other genera including heat-resistant *Bacillales_bacterium_Mi4* (Yamane et al., 2008), halophilic *Salinicola* (Shang et al., 2018) and heterotroph *Petrimonas* (Qian et al., 2020) had a small increase, reaching 4.88, 3.22, and 2.33%, respectively. These three genera could secrete amylase and protease in high temperature environments and promote the degradation of OM. The three genera could also decompose fiber, hemicellulose, lignin and other refractory OM, promoting the degradation of macromolecular OM and maintaining the stability of the heap composting (Yamane et al., 2008; Shang et al., 2018; Qian et al., 2020). However, the abundance of bacteria with ability to adsorb and passivate heavy metals, such as *Idiomarina*, *Bacillus*, *Geobacter*, and *Pseudomonas*, decreased to 1.92, 1.01, 0.43, and 0.42%, respectively. This might be due to the poor adaptability of these bacteria to high temperature environments (Zhou et al., 2020). At the cooling stage (WN24d), the abundances of *Tuberibacillus* and *Bacillales_bacterium_Mi4* continued to significantly increase. *Tuberibacillus* was the dominant bacterium, accounting for 44.81%, followed by *Bacillales_bacterium_Mi4*, the second dominant bacterium, accounting for 18.01%. The petroleum-degrading bacterium *Alcanivorax* showed a slight increase (Ramasamy et al., 2020), accounting for 4.93%, while the other bacteria only accounted for a small proportion, and the species composition at the cooling stage was relatively simplified. In summary, the main microorganisms such as *Tuberibacillus*, *Bacillus*, *Geobacter*, *Idiomarina*, *Lactobacillus*, and *Pseudomonas* with heavy metal passivation and OM removal could be enriched during the composting process. At the heating stage, the dominant bacteria such as *Bacillus*, *Geobacter*, *Lactobacillus*, and *Pseudomonas* were enriched. As composting proceeded, the abundance of *Tuberibacillus* increased significantly and became the most dominant bacterium at the heating stage. Compared with other bacteria, *Tuberibacillus* exhibited a better heat-resistance activity, so it became the dominant bacterium in the high temperature period and maturity period of composting. Based on the above results, it was found that the inoculation of composting bacteria such as *Tuberibacillus*, *Bacillus*, *Geobacter*, *Idiomarina*, *Lactobacillus*, and *Pseudomonas* first promoted the process of sludge composting (increasing the temperature), and then the heat-resistance bacteria such as *Tuberibacillus* gradually became the dominant bacteria under high temperature conditions, and further promoted the performance of sludge composting, so that the detoxification of heavy metals might be enhanced during the process of composting.

#### Function prediction of microbial community during composting

**FIGURE 6:**
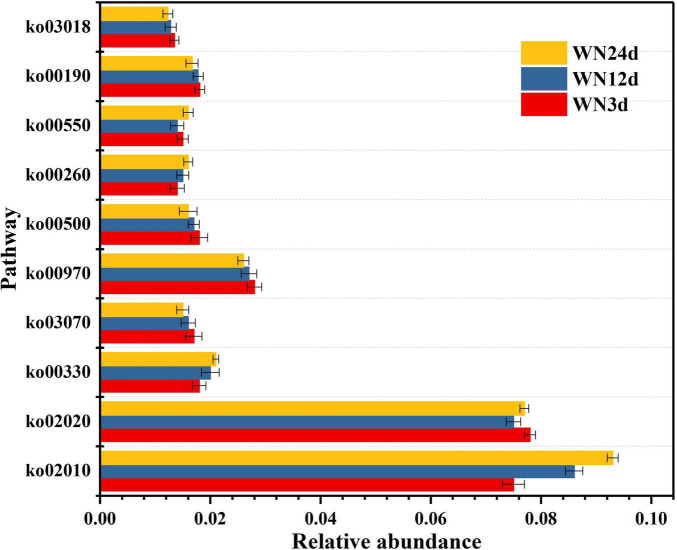
Comparison of differences in abundance of metabolic pathway genes during the composting process (the top 10 metabolic pathways with the largest abundance differences).

The functional changes in the microbial community were predicted by the Tax4Fun method during the composting process, and the results are shown in Figure 6 and Table 4. Clearly, the abundance of functional genes in the same metabolic pathway varied significantly at different composting stages owing to the continuous succession of different dominant bacteria. Take the top 10 metabolic pathways with the greatest abundance differences at different composting stages. During the composting process, the activities of ko02010, ko00330, and ko00260 were the most significant during the cooling stage, were weakened at the thermophilic stage, and finally were inhibited at the heating stage, indicating that with composting processing, the metabolism, amino acid metabolism and membrane transport functions of composting microorganisms gradually became more active. The activities of ko02020 and ko00550 were greater during the heating stage and the cooling stage, but were inhibited at the thermophilic stage, indicating that the extracellular electron transport function of composting microorganisms is inhibited at the thermophilic stage. However, they became more active during the heating stage and the cooling stage owing to the lower temperature, suggesting that the reduction and passivation of heavy metals mainly occurred during the heating stage and the cooling stage. The activities of ko03070, ko00970, ko00500, ko00190, and ko03018 were the most active during the heating stage, and then gradually decreased during the thermophilic stage, and were inhibited during the cooling stage. This indicated that as the composting time proceeded, the carbohydrate metabolism activity of compost microorganisms decreased gradually.

**TABLE 4:** List of metabolic pathway functions.

Pathway	Metabolic pathway function
ko02010	Environmental information processing; membrane transport; ABC transporters
ko02020	Environmental information processing; signal transduction; two-component system
ko00330	Metabolism; amino acid metabolism; arginine and proline metabolism
ko03070	Environmental information processing; membrane transport; bacterial secretion system
ko00970	Genetic information processing; translation; aminoacyl-tRNA biosynthesis
ko00500	Metabolism; carbohydrate metabolism; starch and sucrose metabolism
ko00260	Metabolism; amino acid metabolism; glycine, serine and threonine metabolism
ko00550	Metabolism; glycan biosynthesis and metabolism; peptidoglycan biosynthesis
ko00190	Metabolism; energy metabolism; oxidative phosphorylation
ko03018	Genetic information processing; folding, sorting and degradation; RNA degradation

## Conclusion

The sludge of treat 2 reached composting maturity after 26 days of co-composting with spent mushroom and spent bleaching. Among the three treatments, treat 2 achieved the best composting performance, and the physicochemical properties of treat 2 were more conducive to the passivation of heavy metals and the oxidation of OMs than other treatments. OM content, EC, pH, and GI of treat 2 were all in line with the standard limit of agricultural sludge. The performance of passivating heavy metals satisfied standard limit for agricultural sludge, which were superior to those of treat 1 and treat 3. The diversity of microbial communities in treat 2 decreased during the composting process. Extensive bacteria such as *Bacillus*, *Geobacter*, *Lactobacillus*, and *Pseudomonas*, which possessed the abilities of heavy metal detoxification and organic oxidizing, were dominant in treat 2 at the heating stage. However, *Tuberibacillus* gradually became the most dominant species at the thermophilic stage and the cooling stage, further contributed a lot to OM degradation and heavy metal passivation. Changes in microbial functions varied from microbial community observed in treat 2, subsequently affecting the performances of heavy metal passivation and OMs removal during composting. These findings may provide useful knowledge to improve the composting efficiency in the future.

## Data availability statement

The data presented in the study are deposited in the NCBI repository, BioProject ID: PRJNA877802.

## Author contributions

WH: writing—original draft preparation, methodology, visualization, investigation, and conceptualization. SC: investigation, visualization, and resources. XT: visualization and resources. XL, HP, PM, and ZW: resources. QX: supervision, funding acquisition, resources, and writing—review and editing. All authors contributed to the article and approved the submitted version.

## References

[B1] Awasthi M. K. Chen H. Duan Y. Liu T. Awasthi S. K. Wang Q. (2019). An assessment of the persistence of pathogenic bacteria removal in chicken manure compost employing clay as additive via meta-genomic analysis. *J. Hazard. Mater.* 366 184–191. 10.1016/j.jhazmat.2018.11.108 30528588

[B2] Chen J. Liu Y. Yang Y. Tang M. Wang R. Jiang L. (2020). Bacterial community structure and gene function prediction in response to long-term running of dual graphene modified bioelectrode bioelectrochemical systems. *Bioresour. Technol.* 309:123398. 10.1016/j.biortech.2020.123398 32325382

[B3] Chen J. Yang Y. Liu Y. Tang M. Wang R. Zhang C. (2019). Bacterial community shift in response to a deep municipal tail wastewater treatment system. *Bioresour. Technol.* 281 195–201. 10.1016/j.biortech.2019.02.099 30822640

[B4] Chen R. Ma X. Yu Z. Chen L. Chen X. Qin Z. (2021). Study on synchronous immobilization technology of heavy metals and hydrolyzed nitrogen during pyrolysis of sewage sludge. *J. Environ. Chem. Eng.* 9:106079. 10.1016/j.jece.2021.106079

[B5] Chen Z. Li Y. Peng Y. Ye C. Zhang S. (2021). Effects of antibiotics on hydrolase activity and structure of microbial community during aerobic co-composting of food waste with sewage sludge. *Bioresour. Technol.* 321:124506. 10.1016/j.biortech.2020.124506 33310386

[B6] Czeka W. Malińska K. Cáceres R. Janczak D. Dach J. Lewicki A. (2016). Co-composting of poultry manure mixtures amended with biochar – The effect of biochar on temperature and C-CO 2 emission. *Bioresour. Technol.* 200 921–927. 10.1016/j.biortech.2015.11.019 26609949

[B7] Ding Y. Xiong J. Zhou B. Wei J. Qian A. Zhang H. (2019). Odor removal by and microbial community in the enhanced landfill cover materials containing biochar-added sludge compost under different operating parameters. *Waste Manag.* 87 679–690. 10.1016/j.wasman.2019.03.009 31109570

[B8] Ezzariai A. Hafidi M. Khadra A. Aemig Q. El Fels L. Barret M. (2018). Human and veterinary antibiotics during composting of sludge or manure: Global perspectives on persistence, degradation, and resistance genes. *J. Hazard. Mater.* 359 465–481. 10.1016/j.jhazmat.2018.07.092 30071464

[B9] Fernández P. M. Viñarta S. C. Bernal A. R. Cruz E. L. Figueroa L. I. C. (2018). Bioremediation strategies for chromium removal: Current research, scale-up approach and future perspectives. *Chemosphere* 208 139–148. 10.1016/j.chemosphere.2018.05.166 29864705

[B10] Guo H. Liu H. Wu S. (2022). Immobilization pathways of heavy metals in composting: Interactions of microbial community and functional gene under varying C/N ratios and bulking agents. *J. Hazard. Mater.* 426:128103. 10.1016/j.jhazmat.2021.128103 34952492

[B11] Jacob J. M. Karthik C. Saratale R. G. Kumar S. S. Prabakar D. Kadirvelu K. (2018). Biological approaches to tackle heavy metal pollution: A survey of literature - ScienceDirect. *J. Environ. Manag.* 217 56–70. 10.1016/j.jenvman.2018.03.077 29597108

[B12] Jordan S. N. Mullen G. J. Murphy M. C. (2008). Composition variability of spent mushroom compost in Ireland. *Bioresour. Technol.* 99 411–418. 10.1016/j.biortech.2006.12.012 17306529

[B13] Li R. Wang Q. Zhang Z. Zhang G. Li Z. Wang L. (2015). Nutrient transformation during aerobic composting of pig manure with biochar prepared at different temperatures. *Environ. Technol.* 36 815–826. 10.1080/09593330.2014.963692 25209736

[B14] Liang D. H. Hu Y. Cheng J. Chen Y. (2021). Effects of various antibiotics on aerobic nitrogen removal and antibiotic degradation performance: Mechanism, degradation pathways, and microbial community evolution. *J. Hazard. Mater.* 422, 126818. 10.1016/j.jhazmat.2021.126818 34390955

[B15] Liao H. Lu X. Rensing C. Friman V. P. Geisen S. Chen Z. (2018). Hyperthermophilic Composting Accelerates the Removal of Antibiotic Resistance Genes and Mobile Genetic Elements in Sewage Sludge. *Environ. Sci. Technol.* 52:266. 10.1021/acs.est.7b04483 29199822

[B16] Liu H. T. Zheng H. X. Chen T. B. Zheng G. D. Gao D. (2014). Reduction in greenhouse gas emissions from sewage sludge aerobic compost in China. *Water Sci. Technol.* 69 1129–1135. 10.2166/wst.2013.773 24647175

[B17] Lu X. Ma X. Qin Z. Chen X. Chen L. Tian Y. (2021). Co-hydrothermal carbonization of sewage sludge and polyvinyl chloride: Hydrochar properties and fate of chlorine and heavy metals. *J. Environ. Chem. Eng.* 9:106143. 10.1016/j.jece.2021.106143

[B18] Mao C. Wang Y. Wang X. Ren G. Yuan L. Feng Y. (2019). Correlations between microbial community and C:N:P stoichiometry during the anaerobic digestion process. *Energy* 174 687–695.

[B19] Mkaa B. Yda B. Ska A. Tao L. A. Hc A. Apc D. (2020). Emerging applications of biochar: Improving pig manure composting and attenuation of heavy metal mobility in mature compost. *J. Hazard. Mater.* 389:122116. 10.1016/j.jhazmat.2020.122116 31972527

[B20] Na Y. K. Kim S. K. Ra C. H. (2021). Evaluation of gamma-aminobutyric acid (GABA) production by Lactobacillus plantarum using two-step fermentation. *Bioprocess. Biosyst. Eng.* 44 2099–2108. 10.1007/s00449-021-02586-8 34032903

[B21] Qian Y. Xu M. Deng T. Hu W. He Z. Yang X. (2020). Synergistic interactions of Desulfovibrio and Petrimonas for sulfate-reduction coupling polycyclic aromatic hydrocarbon degradation. *J. Hazard. Mater.* 407:124385. 10.1016/j.jhazmat.2020.124385 33229269

[B22] Qiu B. Hu Y. Liang C. Wang L. Shu Y. Chen Y. (2020). Enhanced degradation of diclofenac with Ru/Fe modified anode microbial fuel cell: Kinetics, pathways and mechanisms. *Bioresour. Technol.* 300:122703. 10.1016/j.biortech.2019.122703 31911312

[B23] Qiu B. Hu Y. Tang C. Chen Y. Cheng J. (2021). Simultaneous mineralization of 2-anilinophenylacetate and denitrification by Ru/Fe modified biocathode double-chamber microbial fuel cell. *Sci. Total Environ.* 792:148446. 10.1016/j.scitotenv.2021.148446 34465036

[B24] Ramasamy K. P. Rajasabapathy R. Lips I. Mohandass C. James R. A. (2020). Genomic features and copper biosorption potential of a new Alcanivorax sp. VBW004 isolated from the shallow hydrothermal vent (Azores, Portugal). *Genomics* 112 3268–3273. 10.1016/j.ygeno.2020.06.015 32553480

[B25] Rauret G. López-Sánchez J. F. Sahuquillo A. Barahona E. Lachica M. Ure A. M. (2000). Application of a modified BCR sequential extraction (three-step) procedure for the determination of extractable trace metal contents in a sewage sludge amended soil reference material (CRM 483), complemented by a three-year stability study of acetic acid a. *J. Environ. Monit.* 2 228–233. 10.1039/b001496f 11256704

[B26] Shang N. Zhu Q. Dai M. Zhao G. (2018). Complete Genome Sequence of the Heavy-Metal-Tolerant Endophytic Type Strain of Salinicola tamaricis. *Genome Announc.* 6:e318–e358. 10.1128/genomeA.00358-18 29674561 PMC5908926

[B27] Vitali F. Mastromei G. Senatore G. Caroppo C. Casalone E. (2016). Long lasting effects of the conversion from natural forest to poplar plantation on soil microbial communities. *Microbiol. Res.* 182 89–98. 10.1016/j.micres.2015.10.002 26686617

[B28] Wang K. Ma X. C. Yin X. Wu C. Tian Y. (2021). Difference and interplay of microbial communities, metabolic functions, trophic modes and influence factors between sludge and bulking agent in a composting matrix. *Bioresour. Technol.* 336:125085. 10.1016/j.biortech.2021.125085 34049165

[B29] Wang W. Zhai Y. Cao L. Tan H. Zhang R. (2016). Endophytic bacterial and fungal microbiota in sprouts, roots and stems of rice (*Oryza sativa* L.). *Microbiol. Res.* 188–189 1–8. 10.1016/j.micres.2016.04.009 27296957

[B30] Wang X. Selvam A. Chan M. Wong J. (2013). Nitrogen conservation and acidity control during food wastes composting through struvite formation. *Bioresour. Technol.* 147 17–22. 10.1016/j.biortech.2013.07.060 23981269

[B31] Wang X. Zhang J. Chang X. You Q. Zhao S. Zhuo H. (2015). Sludge compost and its application. *Environ. Sci. Manag.* 40 49–52.

[B32] Xu Z. Lu Z. Zhang L. Fan H. Wang Y. Li J. (2021). Red mud based passivator reduced Cd accumulation in edible amaranth by influencing root organic matter metabolism and soil aggregate distribution. *Environ. Pollut.* 275:116543. 10.1016/j.envpol.2021.116543 33556735

[B33] Xu Z. Wang D. Tang W. Wang L. Li Q. Lu Z. (2020). Phytoremediation of cadmium-polluted soil assisted by D-gluconate-enhanced *Enterobacter cloacae* colonization in the Solanum nigrum L. rhizosphere. *Sci. Total Environ.* 732:139265. 10.1016/j.scitotenv.2020.139265 32416401

[B34] Yamane K. Maki H. Nakayama T. Nakajima T. Kitaoka M. (2008). Diversity and Similarity of Microbial Communities in Petroleum Crude Oils Produced in Asia. *Biosci. Biotechnol. Biochem.* 72 2831–2839. 10.1271/bbb.80227 18997416

[B35] Zhang Q. H. Yang W. N. Ngo H. H. Guo W. S. Ao D. (2016). Current status of urban wastewater treatment plants in China. *Environ. Int.* 92–93 11–22. 10.1016/j.envint.2016.03.024 27045705

[B36] Zhou C. Yanzeng L. Yanyan P. Chengsong Y. Shenghua Z. (2020). Effects of antibiotics on hydrolase activity and structure of microbial community during aerobic co-composting of food waste with sewage sludge. *Bioresour. Technol.* 321:124506. 10.1016/j.biortech.2020.124506 33310386

[B37] Zorpas A. A. Lasaridi K. Pociovalisteanu D. M. Loizia P. (2017). Monitoring and Evaluation of Prevention activities regarding household organics waste from insular communities. *J. Clean. Prod.* 172 3567–3577. 10.1016/j.jclepro.2017.03.155

