# Supplementary material for: Microbial community succession in response to sludge composting efficiency and heavy metal detoxification during municipal sludge composting
Source: Front Microbiol. 2022 Oct 5;13:1015949. doi: 10.3389/fmicb.2022.1015949 (PMC9581145; doi:10.3389/fmicb.2022.1015949)
Supplement: Supplementary file 1 [file Data_Sheet_1.docx]

Supplementary material for

Microbial community succession in response to sludge composting efficiency and heavy metal detoxification during municipal sludge composting

Weijiang Han^1,2^; Shuona Chen^1,3^; Xiao Tan^2^; Xin Li^1^; Hua Pan^4^; Peijian Ma^5^; Zhihua Wu^5^; Qilai Xie^1,3*^

1 College of Natural Resources and Environment, South China Agricultural University, Guangzhou 510642, China

2 South China Institute of Environmental Sciences, Ministry of Ecology and Environment, Guangzhou 510655, China

3 Guangdong Provincial Key Laboratory of Agricultural & Rual Pollution Abatement and Environmental Safety, Guangzhou 510642, China

4 Foshan Ecological Environment Bureau Nanhai Branch, Foshan, 528200, China

5 Qingyuan Solid Waste Treatment Center, Qingyuan, 511500, China

*Corresponding author: College of Natural Resources and Environment, South China Agricultural University, Guangzhou 510642, China. E-mail addresses: xieql@scau.edu.cn.

**Table S1 Basic properties of raw and auxiliary materials**

| Material | MC/% | pH | EC/mS·cm^-1^ | Orgnic content/g kg^-1^ | C/N/% | TN/% | GI |
| --- | --- | --- | --- | --- | --- | --- | --- |
| Sludge | 67.67 | 6.34 | 2.9 | 68.25 | 2.58 | 1.87 | 0.30 |
| Spent mushroom | 54.34 | 4.77 | 2.16 | 75.17 | 1.97 | 0.53 | / |
| Spent bleaching earth | 23.59 | 5.17 | 1.15 | 39.00 | 0.36 | 0.16 | / |

**Table S2 Heavy metals contents of raw and auxiliary materials（mg kg^-1^）**

|  | Cd | Cu | Zn |
| --- | --- | --- | --- |
| Sludge | 1.17 | 682.2 | 431.2 |
| Spent mushroom | 1.22 | 13.39 | 62.23 |
| Spent bleaching earth | 0.17 | 24.57 | 247.81 |
